# Supplementary material for: Regulating ferredoxin electron transfer using nanobody and antigen interactions
Source: RSC Chem Biol. 2025 Jan 31;6(5):746–53. doi: 10.1039/d4cb00257a (PMC11886610; doi:10.1039/d4cb00257a)
Supplement: CB-006-D4CB00257A-s001 [file CB-006-D4CB00257A-s001.pdf]

Supplementary Information (SI) for RSC Chemical Biology.
This journal is © The Royal Society of Chemistry 2025

SUPPORTING INFORMATION

Regulating ferredoxin electron transfer using nanobody and antigen interactions

Albert Truong[1,2] and Jonathan J. Silberg[2,3,4*]

Author affiliations:

1. Biochemistry and Cell Biology Graduate Program, Rice University, 6100 Main Street, MS-180, Houston, Texas 77005
2. Department of Biosciences, Rice University, 6100 Main Street, MS-140, Houston, TX, 77005
3. Department of Bioengineering, Rice University, 6100 Main Street, MS-142, Houston, TX, 77005
4. Department of Chemical and Biomolecular Engineering, Rice University, 6100 Main Street, MS-362, Houston, TX, 77005

*Address correspondence to: Jonathan J. Silberg
Biosciences Department
6100 Main Street
Houston, TX 77005 USA
Tel: 713-348-3849
Email: joff@rice.edu

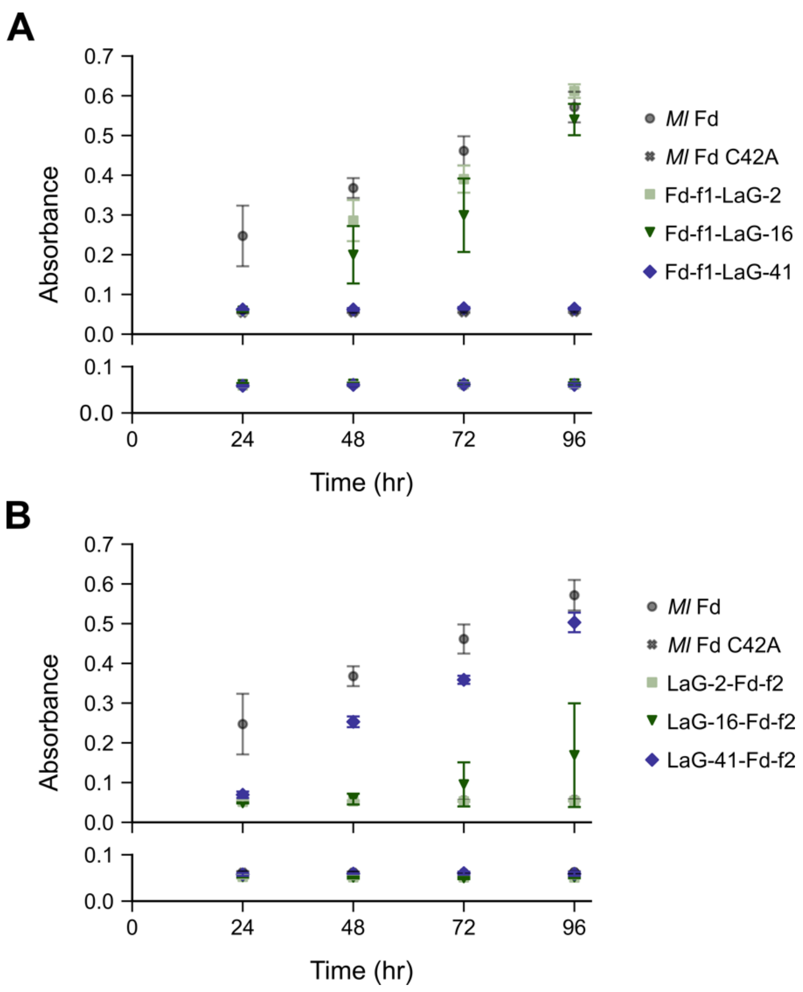

Figure S1. Growth complementation of *E. coli* EW11 in selective medium across a four-day incubation. Growth complementation of the Fd split after residue 35 at different times, including (**A**) three Fd-f1-Nb and GFP-Fd-f2 variants and (**B**) three Fd-f1-GFP and Nb-Fd-f2 variants. Data points represent the mean absorbance values of cultures following 24, 48, 72, and 96 hours of growth in m9sa. The top panel represents growth in the presence of inducers while the bottom represent growth in the absence. Data represents the average of three biological replicates, with error bars representing ±1 standard deviation.

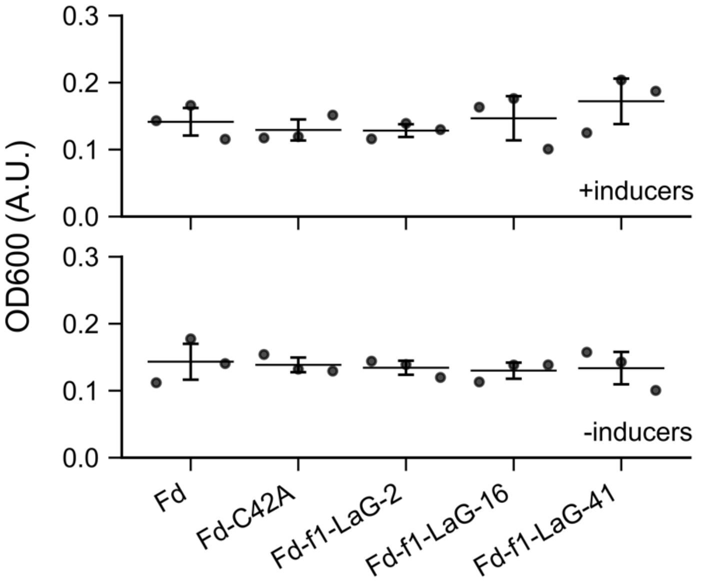

Figure S2. ***E. coli*** **EW11 growth in non-selective medium when expressing sFd-35 fragments having Fd-f1 fused to Nbs and Fd-f2 fused to GFP.** *E. coli* EW11 expressing different Fd-f1-Nb and GFP-Fd-f2 variants (split after residue 35) were grown overnight at 37°C in non-selective m9c media which includes sulfur-containing amino acids. No significant differences ($p > 0.1$, two-tailed Welch's t-test) in optical density are observed between cultures in which expression of split ferredoxin fusions from Figure 1C are induced (*top*) or not induced (*bottom*).

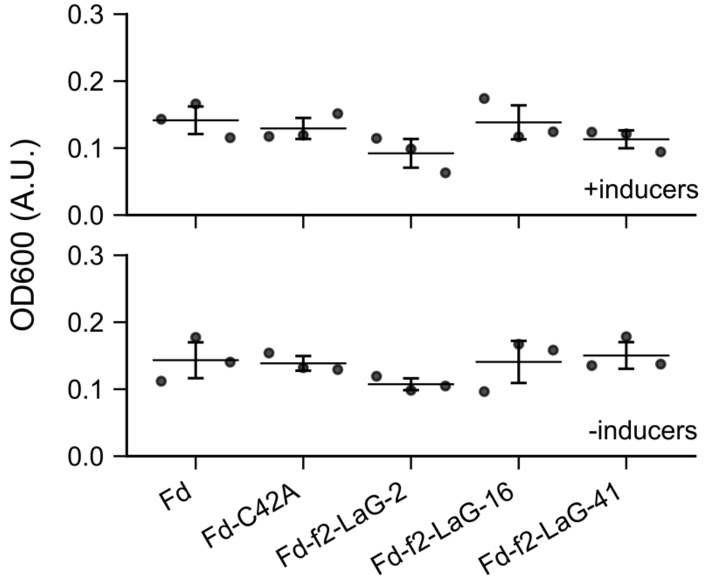

Figure S3. ***E. coli*** **EW11 growth in non-selective medium when expressing sFd-35 fragments having Fd-f1 fused to GFP and Fd-f2 fused to Nbs.** *E. coli* EW11 expressing different Fd-f1-GFP and Nb-Fd-f2 variants (split after residue 35) were grown overnight at 37°C in non-selective m9c media which includes sulfur-containing amino acids. No significant differences ($p > 0.1$, two-tailed Welch's t-test) in optical density are seen between cultures in which expression of split ferredoxin fusions from Figure 1D are induced (*top*) or not induced (*bottom*).

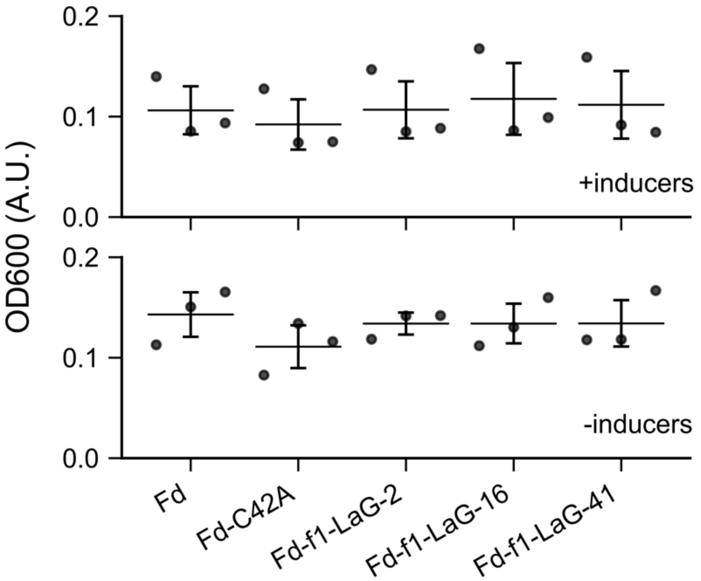

Figure S4. ***E. coli*** **EW11 growth in non-selective medium when expressing sFd-55 fragments having Fd-f1 fused to Nbs and Fd-f2 fused to GFP.** Cells expressing different Fd-f1-Nb and GFP-Fd-f2 variants were grown overnight at 37°C in non-selective medium which includes sulfur-containing amino acids. No significant difference in optical density ($p > 0.1$; two-tailed Welch's t-test) are observed between cultures expressing the variants from Figure 2A in the presence (*top*) or absence (*bottom*) of inducers.

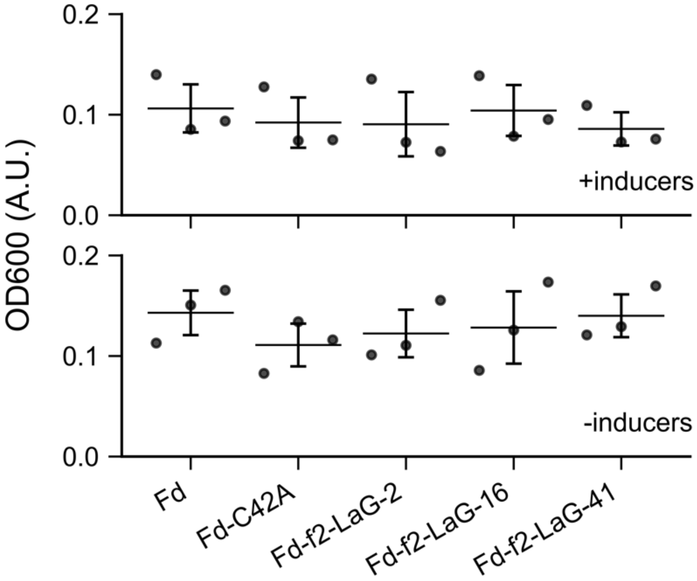

Figure S5. *E. coli* EW11 growth in non-selective medium when expressing sFd-55 fragments having Fd-f1 fused to GFP and Fd-f2 fused to Nbs. Cells expressing different Fd-f1-GFP and Nb-Fd-f2 variants were grown overnight at 37°C in non-selective m9c medium which includes sulfur-containing amino acids. No significant differences in optical density are observed between expressing the split Fd fusions from Figure 2B in the presence (*top*) or absence (*bottom*) of inducers ($p > 0.05$, two-tailed Welch's t-test).

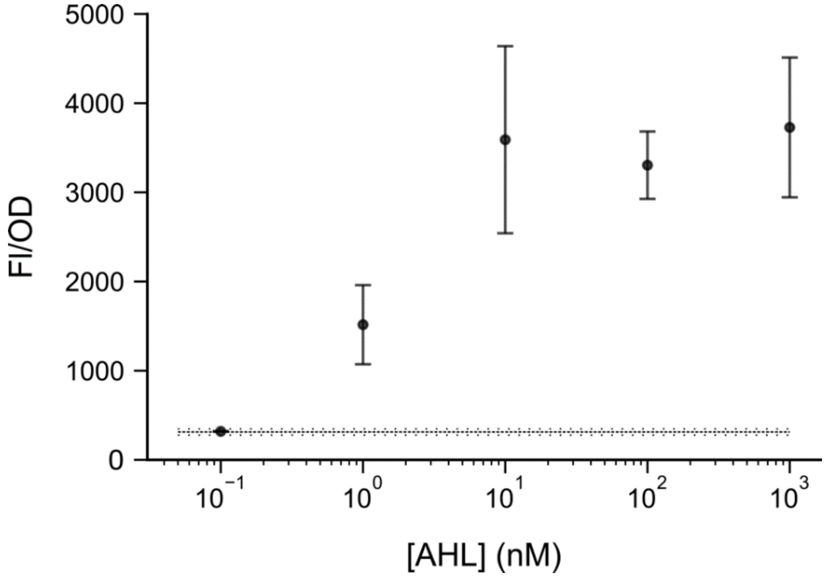

Fig. S6. GFP induction curve. *E. coli* EW11 harboring the vector for GFP expression (pAT019) were induced with 0, 0.1, 1, 10, 100, and 1000 nM AHL in non-selective M9c medium. After 20 hours at 37°C, fluorescence (FI) to optical density (OD) were measured and the ratio was calculated. The data represent the mean from three biological replicates, while error bars represent ±1 standard deviation. The line represents the FI/OD ratio for cells grown in the absence of inducer.

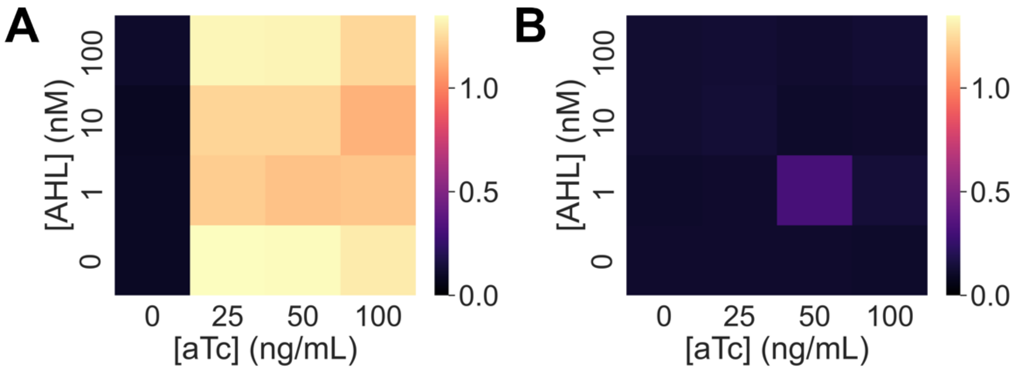

Fig. S7. Effect of inducing GFP expression on Fd complementation. GFP was co-expressed with (**A**) native Fd and (**B**) a Fd-C42A mutant. *E. coli* EW11 were incubated at 37°C for 48 hours in selective medium containing varying concentrations of aTc (0, 25, 50, and 100 ng/mL) and AHL (0, 1, 10, and 100 nM), which induce expression of the Fds and GFP, respectively. Growth complementation was not observed with Fd in the absence of inducer, while all other induction conditions presented similar growth complementation. Data represent the mean of three biological replicates.

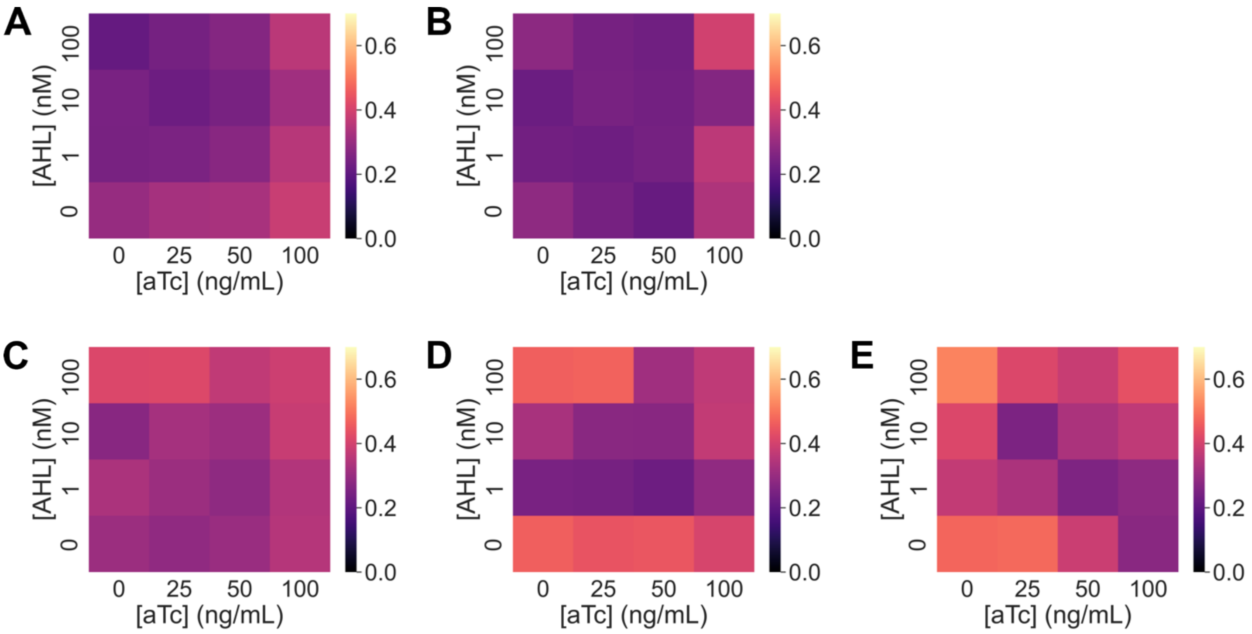

Fig. S8. Growth of cells expressing domain insertion variants under non-selective conditions. GFP was co-expressed with (**A**) native Fd, (**B**) Fd-C42A, (**C**) Fd-LaG-2, (**D**) Fd-LaG-16, and (**E**) Fd-LaG-41 in *E. coli* EW11. *E. coli* EW11 were incubated at 37°C for 20 hours in non-selective medium containing varying concentrations of aTc (0, 25, 50, and 100 ng/mL) and AHL (0, 1, 10, and 100 nM), which induce expression of the Fds and GFP, respectively. All experiments were performed using three biological replicates.

Table S1. Plasmids used in this study. Listed for each plasmid are the name, antibiotic resistance marker (AbR), origin of replication (ori), promoters (P1 and P2) used to regulate protein expression, and proteins expressed (Protein #1 and #2). For protein fusions, the linker sequences (Linker #1 and #2) are provided. n/a, not applicable.

Name	AbR	ori	P1	Protein #1	Linker #1	P2	Protein #2	Linker #2
pFd007	CmR	ColE1	Ptet	Ml-Fd (1-99)	n/a	n/a	n/a	n/a
pFd007-C42A	CmR	ColE1	Ptet	Ml Fd-C42A	n/a	n/a	n/a	n/a
pSH001	KanR	ColE1	PlasR	GFP mut3b	n/a	n/a	n/a	n/a
pSAC01	SmR	p15A	Pcon	Zea mays FNR	n/a	Pcon	Zea mays SIR	n/a
pAT019	KanR	pSC101	PlasR	GFP mut3b	n/a	n/a	n/a	n/a
pRAP007.35	CmR	ColE1	Ptet	Fd(1-35)::FKBP	(GGGGS)$_2$AAA	Plac	Fd(36-99)::FRB	(GGGGS)$_2$AAA
pAT020	CmR	ColE1	Ptet	Fd(1-35)::LaG-2	(GGGGS)$_2$AAA	Plac	Fd(36-99)::GFP	(GGGGS)$_2$AAA
pAT021	CmR	ColE1	Ptet	Fd(1-35)::GFP	(GGGGS)$_2$AAA	Plac	Fd(36-99)::LaG-2	(GGGGS)$_2$AAA
pAT022	CmR	ColE1	Ptet	Fd(1-35)::LaG-16	(GGGGS)$_2$AAA	Plac	Fd(36-99)::GFP	(GGGGS)$_2$AAA
pAT023	CmR	ColE1	Ptet	Fd(1-35)::GFP	(GGGGS)$_2$AAA	Plac	Fd(36-99)::LaG-16	(GGGGS)$_2$AAA
pAT024	CmR	ColE1	Ptet	Fd(1-35)::LaG-41	(GGGGS)$_2$AAA	Plac	Fd(36-99)::GFP	(GGGGS)$_2$AAA
pAT025	CmR	ColE1	Ptet	Fd(1-35)::GFP	(GGGGS)$_2$AAA	Plac	Fd(36-99)::LaG-41	(GGGGS)$_2$AAA
pAT026	CmR	ColE1	Ptet	Fd(1-55)::LaG-2	(GGGGS)$_2$AAA	Plac	Fd(56-99)::GFP	(GGGGS)$_2$AAA
pAT027	CmR	ColE1	Ptet	Fd(1-55)::GFP	(GGGGS)$_2$AAA	Plac	Fd(56-99)::LaG-2	(GGGGS)$_2$AAA
pAT028	CmR	ColE1	Ptet	Fd(1-55)::LaG-16	(GGGGS)$_2$AAA	Plac	Fd(56-99)::GFP	(GGGGS)$_2$AAA
pAT029	CmR	ColE1	Ptet	Fd(1-55)::GFP	(GGGGS)$_2$AAA	Plac	Fd(56-99)::LaG-16	(GGGGS)$_2$AAA
pAT030	CmR	ColE1	Ptet	Fd(1-55)::LaG-41	(GGGGS)$_2$AAA	Plac	Fd(56-99)::GFP	(GGGGS)$_2$AAA
pAT031	CmR	ColE1	Ptet	Fd(1-55)::GFP	(GGGGS)$_2$AAA	Plac	Fd(56-99)::LaG-41	(GGGGS)$_2$AAA
pAT032	CmR	ColE1	Ptet	Fd(1-35)::LaG-2	(GGGGS)$_1$AAA	Plac	Fd(36-99)::GFP	(GGGGS)$_2$AAA
pAT033	CmR	ColE1	Ptet	Fd(1-35)::LaG-2	(GGGGS)$_2$AAA	Plac	Fd(36-99)::GFP	(GGGGS)$_1$AAA
pAT034	CmR	ColE1	Ptet	Fd(1-35)::LaG-2	(GGGGS)$_1$AAA	Plac	Fd(36-99)::GFP	(GGGGS)$_1$AAA
pAT035	CmR	ColE1	Ptet	Fd(1-35)::LaG-2	(GGGGS)$_3$AAA	Plac	Fd(36-99)::GFP	(GGGGS)$_2$AAA
pAT036	CmR	ColE1	Ptet	Fd(1-35)::LaG-2	(GGGGS)$_2$AAA	Plac	Fd(36-99)::GFP	(GGGGS)$_3$AAA
pAT037	CmR	ColE1	Ptet	Fd(1-35)::LaG-2	(GGGGS)$_3$AAA	Plac	Fd(36-99)::GFP	(GGGGS)$_3$AAA
pAT038	CmR	ColE1	Ptet	Fd(1-35)::GFP	(GGGGS)$_1$AAA	Plac	Fd(36-99)::LaG-2	(GGGGS)$_1$AAA
pAT039	Cm	ColE1	Ptet	Fd(1-35)::LaG-16	(GGGGS)$_3$A	Pla	Fd(36-99)::GFP	(GGGGS)$_3$A

	R				AA	c		AA
pAT040	Cm R	ColE1	Ptet	LaG-2 inserted Fd	(GGGGS)$_2$A AA	n/a	n/a	n/a
pAT041	Cm R	ColE1	Ptet	LaG-16 inserted Fd	(GGGGS)$_2$A AA	n/a	n/a	n/a
pAT042	Cm R	ColE1	Ptet	LaG-41 inserted Fd	(GGGGS)$_2$A AA	n/a	n/a	n/a
